# Pulmonary manifestations of grade III lymphomatoid granulomatosis complicated by haemophagocytic lymphohistiocytosis: Rare disorders

**DOI:** 10.1002/jha2.240

**Published:** 2021-06-15

**Authors:** Claudette Phillips, Ayoma D. Attygalle, Sunil Iyengar, Andrew Wotherspoon, David Cunningham, Bhupinder Sharma

**Affiliations:** ^1^ Royal Marsden Hospital NHS Trust London UK; ^2^ Institute of Cancer Research London UK

A 58‐year‐old female without past medical history presented with dyspnoea and reduced exercise tolerance. Computed tomography (CT) showed multifocal mid/lower lung consolidative/ground glass lesions, fluorodeoxyglucose (FDG)‐avid on positron emission tomography (PET); right image.


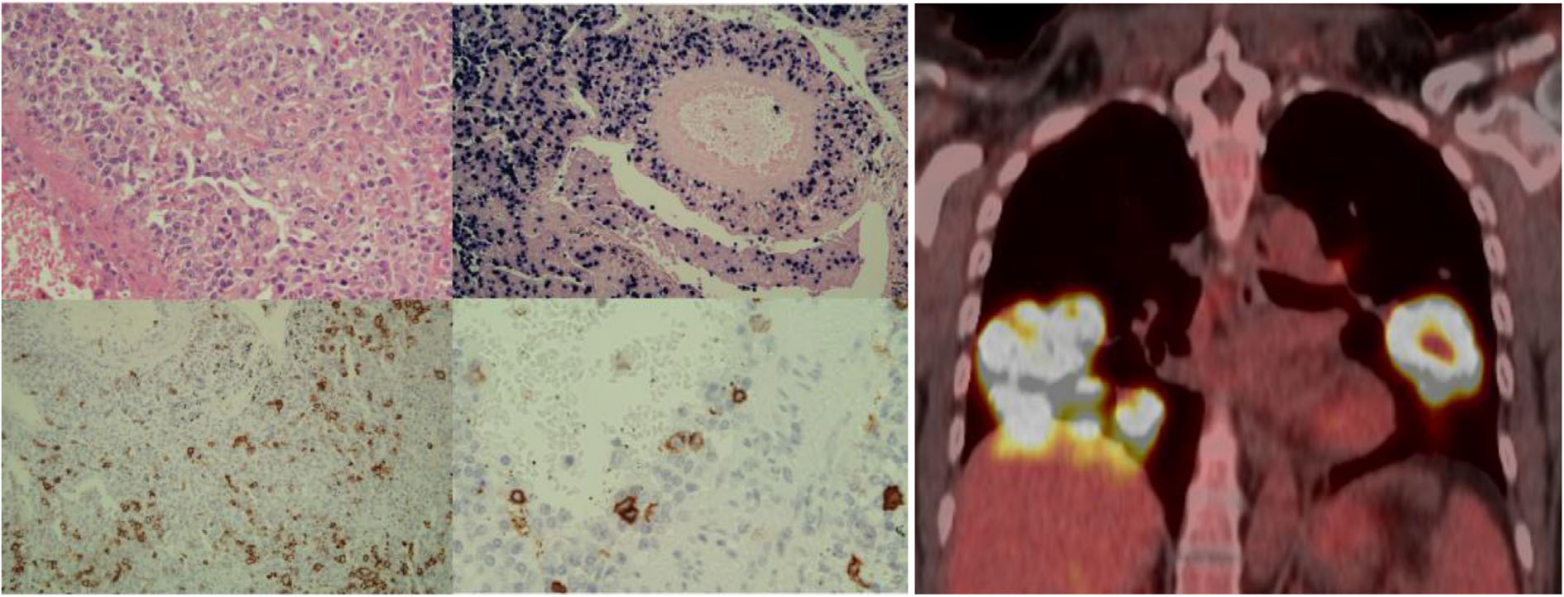


Diagnosis was elusive, with worsening symptoms corticosteroids were commenced and biopsy planned. Ground glass infiltrate improved (on CT) with steroids, thus, while the differential for consolidation is wide, working diagnoses leaned toward lymphoproliferative disorders (LPDs), which cause the entire myriad of lung radiological appearances.

Biopsy identified pleomorphic lymphoid infiltrate (top right) positive for Epstein‐Barr Encoding Region in‐situ hybridisation (top left). Staining for CD20 antigen highlighted a large cell population (bottom left) seen to infiltrate a blood vessel wall (bottom right). Lymphomatoid granulomatosis (LYG) Grade III, a rare Epstein‐Barr virus driven extranodal angiocentric, angiodestructive LPD, was diagnosed.

The aetiology is poorly understood but immunodeficiency is a factor. The lungs are the commonest organ involved at presentation (>90% cases). Pulmonary opacities result from lymphoid infiltrates with a prominent inflammatory response, often centrally necrosing, causing cavitation. Other common involved sites include skin, brain, kidneys, and liver; nodal and splenic involvement are very rare.

The patient underwent a multiply relapsing course. At the first relapse, LYG manifest with secondary haemophagocytic lymphohistiocytosis: a rare condition with uncontrolled lymphocyte and macrophage proliferation, massive cytokine release and organ infiltration (patient presenting with fever, hepatosplenomegaly, pancytopenia, hyperfibrinolysis).

Several regimens were required to reach remission: cyclophosphamide, doxorubicin, prednisolone, rituximab, vincristine (R‐CHOP); rituximab, gemcitabine, cisplatin and methylprednisolone (R‐Gem‐P); rituximab, prednisolone, cyclophosphamide, etoposide, bleomycin, vincristine, methotrexate (R‐PCE2BOM); lomustine, etoposide, cytarabine, melphalan (LEAM) conditioned autologous stem cell transplant (ASCT).

Post‐ASCT PET demonstrated lung uptake, and biopsy suggested necrotic lymphoma, lenalidomide treatment was planned; however interim PET (without treatment) demonstrated regression of uptake.

It is vital to appreciate response assessment is challenging in lung involvement by LPDs: “FDG uptake” will reflect a Deauville Score of “*X*” (*activity unrelated to viable lymphoma*) in a proportion of cases, and PET is not prognostic.

The patient remains in remission 41 months post‐ASCT.

## CONFLICT OF INTEREST

The authors declare that there is no conflict of interest that could be perceived as prejudicing the impartiality of the research reported.

